# Serological, pathological, and scintigraphic assessment of *Hemiscorpius lepturus* effects on renal dysfunction in rats 

**DOI:** 10.22038/ijbms.2018.31426.7585

**Published:** 2018-12

**Authors:** Ali Movahed, Hossein Fatemikia, Kaveh Tanha, Abdolhamid Esmaili, Euikyung Kim, Nasser Mohammadpour Dounighi, Soodabeh Zendeboodi, Ramin Seyedian

**Affiliations:** 1 The Persian Gulf Tropical Research Center, Biochemistry Group, Bushehr University of Medical Sciences, Bushehr, Iran; 2 Departments of Physiology, School of Medicine, Shiraz University of Medical Sciences, Shiraz, Iran; 3 The Persian Gulf Nuclear Medicine Research Center, Bushehr University of Medical Sciences, Bushehr, Iran; 4 Department of Pathology, Bushehr University of Medical Sciences, Bushehr, Iran; 5 College of Veterinary Medicine, Gyeongsang National University, Jinju, South Korea; 6 Department of Human Vaccine and Serum, Razi Vaccine and Serum Research Institute, Agricultural Research, Education and Extension Organization, Karaj, Iran; 7 Department of Nephrology, Bushehr University of Medical Sciences, Bushehr, Iran; 8 Department of Pharmacology, Bushehr University of Medical Sciences, Bushehr, Iran

**Keywords:** Envenomation, Fibrin thrombi, Hemiscorpius lepturus, Kidney, Scorpion, Scintigraphy

## Abstract

**Objective(s):**

*Hemiscorpius lepturus* is one of the dangerous scorpions of Iran leading to acute kidney injury (AKI) especially in infants. The purpose of this animal study was to compare the serological, pathological and scintigraphic data to quickly predict the occurrence of this disorder.

**Materials and Methods:**

In two groups of animals, each contained five rats, *H. lepturus *venom (1200 µg/Kg) were injected intravenously via the tail vein. At three hours and one week later, ^99m^ Tc-DMSA (3 mCi) was intravenously injected and renal scintigraphy was performed after an hour. Moreover, plasma levels of creatinine, sodium, potassium, and blood urea nitrogen (BUN) were measured. At the end of the study, renal tissues were excised and prepared to perform pathological evaluation after Hematoxylin and Eosin staining.

**Results:**

All serological indices were remained unchanged compared to control. A large number of glomerular fibrin thrombi with entrapped red blood cells and simplified tubular epithelium in dilated and ectatic tubules were observed in high power field (×100) four hours after envenomation, which reduced significantly one week later. In our scintigraphic study, there was a statistically significant difference (*P*<0.05) in kidney count rate per pixels (CRPP) in both acute and chronic phases compared to the sham group that received normal saline (0.84±0.05 and 1.36±0.07 versus 1.7±0.05).

**Conclusion:**

The results of this preliminary animal study suggest renal scintigraphy is a non-invasive method to predict the occurrence of the AKI in *H. lepturus* envenomation. It leads the way for more investigation to counteract the renal failure induced by this venom.

## Introduction

Scorpions with about 2000 known species are apparently much less compared to other insects and spiders (1). The scorpion sting is common in tropical and subtropical areas of the world, including southwestern part of Iran, especially in Khuzestan province (2). Interestingly, only six species are dangerous in this area, including *Hemiscorpius lepturus* known as Gadim (the tail of cow) (3, 4). The painless sting, hemoglobinuria, hematuria and cutaneous reactions are orchestrated by this kind of scorpion (5). Importantly, in few cases, envenomation causes massive hemolysis leading to kidney tubular necrosis, hemolytic uremic syndrome and disseminated intravascular coagulation, especially in infants. Therefore, it requires effective therapeutic interventions, particularly when the physicians encounter with the central sting (6, 7). It is worth noting that the kidney has the highest accumulation rate of the radiolabeled venom following subcutaneous injections in rats (8). The nephrotoxic properties of this venom have been shown in mice making it necessary for finding the most sensitive scanning method to predict time and extent of the injury (9). Renal scintigraphy with Technetium-99m-dimercaptosuccinic acid (^99m ^Tc-DMSA) is a new technique recently being used to evaluate the renal tubular function, probably, due to the accumulation of the DMSA in the proximal tubular cells (10). So far, according to the previous investigations, there has been no consensus about the minimal dose, route of injection and finally, the time of renal pathological deformities provoked by the venom in laboratory animals. At first, the purpose of this study was to delineate renal pathological abnormalities following envenomation in acute and chronic phases in detail. Secondly, the scintigraphic results were in line with pathological analysis. It is notable that this animal study was performed for the first time to predict acute kidney injury (AKI) as soon as possible to prevent the irreversible and destructive phenomenon following envenomation.

## Materials and Methods

***Scorpion venom***

*H. lepturus* venom was obtained from Razi Institute of Iran (Hesarak, Karaj). The scorpions were captured from Khuzestan province and direct electrical shock (15V) was conducted to their telsons. The pooled venom was lyophilized and stored at -20ºC until analysis. At the time of the experiment, the samples were reconstituted with dilution in normal saline.

All experimental procedures were approved by the Ethical Committee for animal care of the Bushehr University of Medical Sciences (IR.BPUMS.REC.1397.037).

***Experimental protocol***

Male Wistar rats (200-250 g) were obtained from animal house of Bushehr University of Medical Sciences. They were housed in polycarbonate cages (three animals in each). Animals were kept at a room temperature of 23± 2 °C and 12-hr dark light cycle started at 7:00 AM. 

Rats (n=5 in each group) were anesthetized with intraperitoneal injection of ketamine hydrochloride (100 mg/kg) and xylazine hydrochloride (10 mg/kg). *H. lepturus *venom (400 μg/400 μl) was injected via tail vein in two groups, within two minutes with an infusion pump (Programmable Double Syringe Pump, New Era Pump System, United States). Normal saline was instilled intravenously in the control groups (acute and chronic) with the same schedule. 

^99m^Tc-DMSA (3 mCi) was intravenously injected from the same route three hours after venom injection, and renal scintigraphy with two opposed planar images (anterior and posterior) was collected an hour later. This process was repeated one week later for the third group. Radionuclide imaging was performed with the HiReSPECT imaging system (Parto Negar Persia Co, Iran), a small-animal SPECT system equipped with two gamma camera fitted with parallel-hole collimators (11, 12).

The region of interest (ROI) was selected manually for the kidneys and after background correction, the amount of renal uptake was calculated as the geometric mean of the counts within the selected ROIs in the ventral and the dorsal images. Total amount of the DMSA uptake was expressed as the count-rate-per-pixel (CRPP) ratio, which was calculated as the sum of both kidneys counts divided by the number of the pixels within the ROIs for each kidney.

***Serological and pathological analyses***

Blood samples were collected from the sacrificed animals with a heparinized syringe. 

The plasma samples were separated by centrifugation at 5000 rpm for 10 min. Plasma creatinine, urea, sodium, and potassium levels were measured using the EasyLyte analyzer. Removed kidneys were fixed in 10% formalin solution and transferred to the laboratory. They were embedded in paraffin and cut in five-micrometer section, deparaffinized and finally stained with Hematoxylin-Eosin. The samples were approached for glomerular changes, tubular dilatation and finally moderate to severe necrosis. Each kidney slide was examined in 5 fields for severity of changes on a scale of mild (+1), moderate (+2) and severe (+3) categories. 

***Statistical analysis***

Statistical analyses were performed using SPSS 16.0. Continuous variables were expressed as the mean ±standard deviation. Mann-Whitney tests were used to compare the variables before and after envenomation in acute and chronic phases. The significance was accepted at *P*<0.05. 

## Results

***Biochemical assays***

There was no animal death during our experiment due to envenomation. No changes were found in serum creatinine and BUN in any group. Plasma sodium and potassium levels had no major alterations in the groups (Table 1). 

***Pathological results***

**Figure 1:**
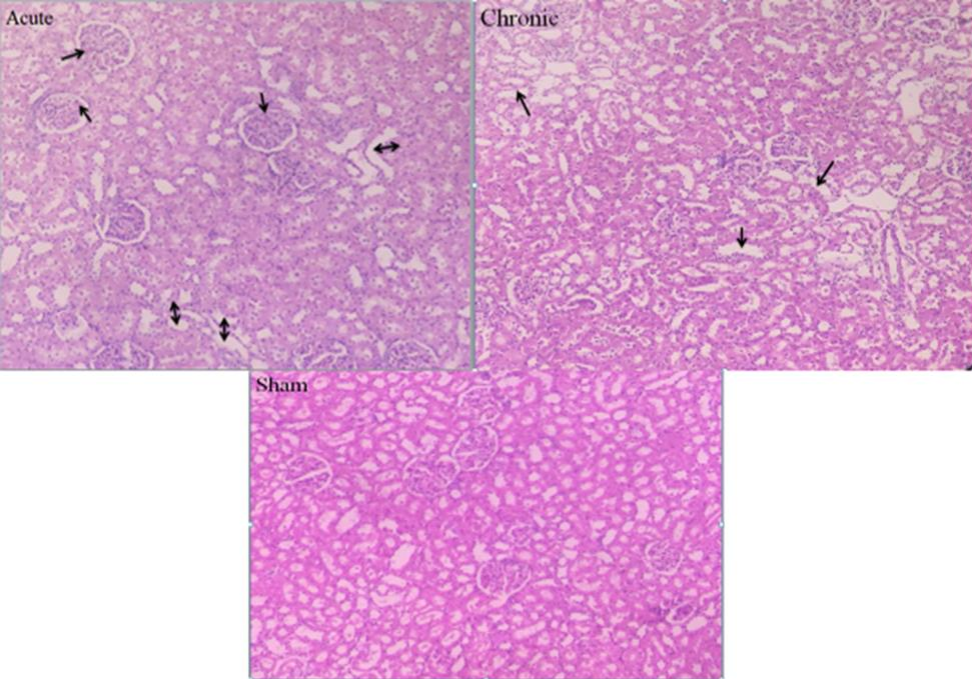
Photomicrograph of the kidney tissues of rats in our groups (H&E .20X). Glomerular complex and renal tubular apparatus are normal in the sham group. Cytoplasmic vacuolization and loss of brush borders are observed in chronic phase (arrows). A fairly large number of fibrin thrombi and simplified tubules are observed in the acute phase (arrows)

Light microscopic study of the renal cortex showed morphological alterations in envenomed groups compared to the control (Figure 1). 

Dilated and simplified proximal tubules and fibrin thrombi in glomerular apparatus were clear in low power magnification in envenomed rats.

**Figure 2:**
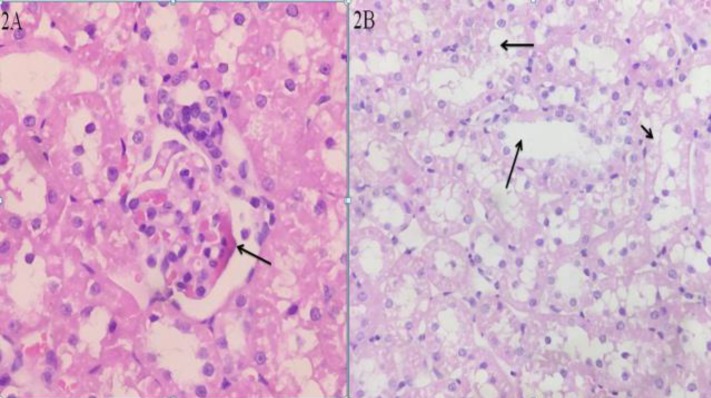
Glomerular injuries with *Hemiscorpius lepturus* venom injection in rats. Representative photomicrograph stained with Hematoxylin and Eosin (2A). The kidney from a rat belonged to acute phase showing glomerular fibrin thrombi and entrapped red blood cells (arrow). Tubular injuries with *H. lepturus *envenomation is shown in Figure 2B at Original magnification ×100. Ectatic and simplified proximal tubules are observed in the acute phase (arrows)

On the contrary to the control group, diffuse pathological abnormalities including glomerular fibrin thrombi with entrapped red blood cells and endothelial swelling were observed in the renal cortical segments four hours following venom injection in high power field (Figure 2A). Another promising finding was deterioration in proximal tubules including simplified epithelium and atypical nuclear reaction proposing acute tubular necrosis four hours after envenomation)Figure 2B(.

Additionally, thinning and the absence of brush borders were observed at this stage.

Finally, in the chronic phase, the fibrin thrombi were observed in few glomeruli accompanied by simplified epithelium in dilated proximal tubules. These abnormalities were less prominent than acute phase (Table 2).

 ***Scintigraphic results***

**Figure 3:**
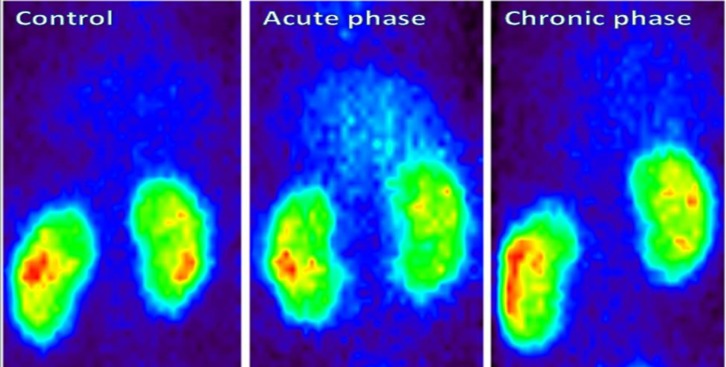
Renal scintigraphic color images of ^99m^Tc-DMSA uptake in three groups of animals. Background activity was more than control in the acute phase

The ^99m^-TC radiolabeling was performed according to the manufacturer’s instruction. Gama scintigraphic color images of the ^99m^Tc-DMSA uptake in our groups are demonstrated in Figure 3, while the amounts of CRPP (kidney count per pixel) as the renal uptake of radiotracer is shown in Table 1. Background activity increased in the acute phase (4 hr after venom injection) compared to the other groups (Figure 3). The CRPP decreased significantly in acute and chronic phases as compared to the control (0.84±0.05 and 1.36±0.07 versus 1.7±0.05). 

## Discussion

Ischemia, sepsis and nephrotoxic drugs are defined as the main causes of AKI with a poor prognosis. Meanwhile, in developing countries, this threatening disease is generally found in rural areas secondary to diarrhea, poor obstetric cases and finally envenomation. The current dataset comprises of 52 species of the scorpions, which mostly exist in Iran as compared to other areas (13). Prevention of the AKI due to envenomation with *Hemiscorpius lepturus *is one of the most important attempts for the envenomed patients. Generally, there is no common consensus on the time and method of treatment of the envenomed patients to predict AKI (14, 15). It seems that introduction of ^99m^TC-DMSA scintigraphy as a non-invasive test to determine the kidney deterioration in envenomed rats may develop new approach for early and accurate diagnosis of this threatening disease (16). In this study, plasma creatinine and BUN levels, as two insensitive and inexpensive laboratory indices, were measured to determine the renal failure. Based on the results, there were no significant changes in poisoned and control animals regarding these two parameters in our groups making the diagnosis of kidney failure impossible at the definite time (Table 1). In a parallel study, serum sodium and potassium levels did not change in envenomed rats, making the diagnosis of electrolyte disturbances and cell rupture questionable. Our results are in agreement with previous studies wherein subcutaneous injection of the venom (10 µg) had no effects on azotemia or alterations in serum potassium levels in rats even twenty-four hours later (17). Reduction of hemoglobin, the occurrence of microalbuminuria and production of ghost cells were highlighted in our previous reports in envenomed rats (5). In another study, one lethal dose (LD_50_) of *H. lepturus* venom (5 mg/kg) was injected by different routes (Intradermal, subcutaneous and intramuscular) to define the pathological changes in internal organs including kidney in mice (9). Disorganized glomeruli and necrotic tubular changes were produced 3 hr and reached the maximum level at 6 hr after envenomation. There were no significant changes in biomarkers like lactate dehydrogenase and creatinine phosphokinase, four hours after venom injection. In another experiment, subcutaneous injection of this venom at dose levels of 500, 1000 and 1500 mg/kg provoked elevation of serum creatinine and BUN three hours following envenomation in rabbits (18). It is worth noting that there was no arrhythmia following intravenous injection of *H. lepturus* venom (1200 µg/kg) in rats (19). When comparing our results to those of previous experiments, it should be pointed out that there is a genus difference in response to envenomation (rat and mouse versus rabbit). It seems that plasma creatinine and BUN measurements were found to be weak indicators of AKI in our animal experiments (20).

However, we acknowledge that there are controversies among researchers as to find the relationship between dose and the pathological abnormalities in rats. In earlier reports, Pipelzadeh *et al*. stated that subcutaneous injection of the venom (50 µg) caused devastating changes in the tubular cells and glomerular apparatus one week later in rats (21). In another study, disorganization and congestion of the renal cortex were clear 24 hr following intraperitoneal injection (3 µg/g body weight) of this venom (22).

**Table 1:** Analysis of different biochemical parameters and tissue Tc-99m dimercaptosuccinic acid (DMSA) following intravenous injections of *Hemiscorpius Lepturus* venom in rats.

**Groups**	**CRPP**	**Creatinine(mg/dl)**	**BUN(mg/dl)**	**Na** ^+ ^ **(meq/L)**	**K** ^+^ **(meq/L)**
**Acute**	0.84±0.05^*^	0.81±0.14	24.3±0.21	156.2±2.3	4.3± 1.2
**Chronic**	1.36±0.07^*^	0.64±0.21	26.1± 1.2	150.1±2.5	5.2± 2.4
**Control**	1.7±0.05	0.71±0.42	21.3± 1.6	154.3±3.4	4.6± 2.8

Acute: Four hours after envenomation. Chronic: One week after envenomation. Control: Four hours following normal saline injection. CRPP: Kidney count rate per pixel. BUN: Blood Urea Nitrogen. Na^+^: Sodium K^+^: Potassium.

Data are presented as means ± standard deviation (n=5), which were analyzed by

Mann-whitney test. The significance level was set at *P*<0.05

**Table 2:** Pathological numbers in *Hemiscorpius lepturus *evoked nephropathies in our groups following envenomation

*Pathological findings*	Control	Acute	Chronic
***Fibrin thrombi***	**0**	**2+**	**1+**
***Endothelial swelling***	**0**	**3+**	**1+**
***Simplified epithelium***	**0**	**2+**	**1+**
***Nuclear reaction atypia***	**0**	**3+**	**1+**
***Dilated proximal tubules***	**0**	**2+**	**1+**
***Vacuolization of epithelial cell cytoplasm***	**0**	**3+**	**1+**

Sham: Normal saline injection. Acute: Four hours after the injection of venom. Chronic: One week after the injection of venom

In regard to the great differences in doses (50 µg/rat versus 3 µg/g body weight) and time intervals of the onset of pathological deterioration (one week versus 24 hr), the information about the renal damages and the responsible factors are limited. Our pathological results were in line with the latter study since intravenous injection of the *H. lepturus *venom (2 µg/g body weight) could imply dramatic changes including glomerular apparatus and tubular complexes after 4 hr of envenomation. 

Scintigraphy with ^99m^Tc-DMSA is a newly developed method for capturing two-dimensional images from internal organs with external detectors (gamma cameras).

It is noteworthy that there is no general agreement about the mechanism of the absorption and accumulation of DMSA in renal tissues (23). According to the qualitative results (Figure 3), the background activities of the renal scintigraphy in group 2 (four hours following envenomation) was more than other groups. It probably stems from different origins including decrement of renal blood flow, acid-base disorders and proximal tubular cell dysfunction (24). Another promising finding was decreasing the Technetium uptake per renal pixel (CRPP) in the acute and chronic phases of envenomation compared to the control (Table 1). Moreover, it seems that the presence of norepinephrine-like substances in this venom could provoke transient vasoconstriction and reduction of CRPP and increase in the background activity, especially in the acute phase (19). It is better to perform this experiment at different time intervals with adrenergic antagonists like propranolol and prazosin to counteract the detrimental renal effects of *H. lepturus* venom in rats (25). Our scintigraphic experiment was in agreement with pathological results showing reversible proximal tubular disorders.

## Conclusion

The novelty of this animal study was to find the scintigraphic analyses as a non-invasive method to predict the destruction of kidney tissues due to the intravenous venom injection in animal study as early as possible. In addition, for the first time large numbers of fibrin thrombi with ectatic tubules were observed in envenomed rats. At the same time, interestingly, there was no mortality in our groups despite transient cardiovascular changes in our previous study after envenomation.

In summary, findings of this study can be considered as the basis for more investigations to find out a new therapeutic method to counteract the occurrence and complications of AKI due to envenomation in human studies.
